# Experimental and in-host evolution of triazole resistance in human pathogenic fungi

**DOI:** 10.3389/ffunb.2022.957577

**Published:** 2022-08-23

**Authors:** Mariana Handelman, Nir Osherov

**Affiliations:** Department of Clinical Microbiology and Immunology, Sackler School of Medicine, Tel-Aviv University, Tel-Aviv, Israel

**Keywords:** *Aspergillus fumigatus*, antifungal resistance, triazole antifungals, evolution of triazole resistance, serial clinical isolates

## Abstract

The leading fungal pathogens causing systemic infections in humans are *Candida* spp., *Aspergillus fumigatus*, and *Cryptococcus neoformans*. The major class of antifungals used to treat such infections are the triazoles, which target the cytochrome P450 lanosterol 14-α-demethylase, encoded by the *ERG11* (yeasts)/*cyp51A* (molds) genes, catalyzing a key step in the ergosterol biosynthetic pathway. Triazole resistance in clinical fungi is a rising concern worldwide, causing increasing mortality in immunocompromised patients. This review describes the use of serial clinical isolates and *in-vitro* evolution toward understanding the mechanisms of triazole resistance. We outline, compare, and discuss how these approaches have helped identify the evolutionary pathways taken by pathogenic fungi to acquire triazole resistance. While they all share a core mechanism (mutation and overexpression of *ERG11/cyp51A* and efflux transporters), their timing and mechanism differs: *Candida and Cryptococcus* spp. exhibit resistance-conferring aneuploidies and copy number variants not seen in *A. fumigatus*. *Candida* spp. have a proclivity to develop resistance by undergoing mutations in transcription factors (*TAC1, MRR1, PDR5*) that increase the expression of efflux transporters. *A. fumigatus* is especially prone to accumulate resistance mutations in *cyp51A* early during the evolution of resistance. Recently, examination of serial clinical isolates and experimental lab-evolved triazole-resistant strains using modern omics and gene editing tools has begun to realize the full potential of these approaches. As a result, triazole-resistance mechanisms can now be analyzed at increasingly finer resolutions. This newfound knowledge will be instrumental in formulating new molecular approaches to fight the rapidly emerging epidemic of antifungal resistant fungi.

## Introduction

Systemic fungal infections are an emerging and serious public health concern, with ~1.5 million deaths occurring every year (Brown et al., 2012). The most common fungal pathogens are *Candida* spp., *Aspergillus fumigatus*, and *Cryptococcus neoformans*.

Currently, there are three leading families of antifungals used to treat systemic fungal infections in the clinic: polyenes, echinocandins and triazoles (Gintjee et al., 2020). Triazoles are a first-line treatment, inhibiting fungal growth by targeting the cytochrome P450 lanosterol 14-α-demethylase, encoded by the *ERG11/cyp51A* gene, catalyzing a key step in the ergosterol biosynthetic pathway. All genes mentioned in this review are listed in **Table 1**. This review will focus on the evolution of triazole-resistance in serial fungal isolates from infected patients, and *in-vitro* experiments in which a fungus is passaged under increasing antifungal concentrations.

**Table 1:** List of genes mentioned in this review.

Gene name	Species	Gene family (if relevant)	Encoded protein function
*ABC1*	*C. glabrata*	ABC transporters	ABC transporter
*AbcB*	*A. fumigatus*	ABC transporters	ABC transporter
*AbcD*	*A. fumigatus*	ABC transporters	ABC transporter
*AbcJ*	*A. fumigatus*	ABC transporters	ABC transporter
*ADH4*	*C. albicans*		Short–chain alcohol dehydrogenase
*AFR1*	*C. neoformans*	ABC transporters	ABC transporter
*AgcA*	*A. fumigatus*		Mitochondrial inner membrane aspartate/glutamate transporter
*Asg1*	*A. fumigatus*	Transcription factors	Controlling drug transporters
*ASR1*	*C. albicans*		Involved in stress response
*AtrF*	*A. fumigatus*	ABC transporters	ABC transporter
*AVC1*	*C. neoformans*		Regulator of virulence traits and carbon assimilation
*Cas33*	*C. neoformans*	Lipases	involved in formation of the cryptococcal capsule
*CDR1*	*Candida spp*	ABC transporters	ABC transporter
*CDR2*	*Candida spp*	ABC transporters	ABC transporter
*CDR3*	*C. albicans*		Involved in membrane and cell wall integrity
*CDR4*	*C. albicans*	ABC transporters	ABC transporter
*CGR1*	*C. albicans*		Involved in stress response
*CRD2*	*C. albicans*		Involved in stress response
*CRG2*	*C. neoformans*		Regulator of G protein signaling
*CRZ1*	*C. albicans*		Involved in membrane and cell wall integrity
*CTK1*	*C. neoformans*		Protein kinase
*ECM21*	*C. albicans*		Involved in membrane and cell wall integrity
*eIF2A*	*C. neoformans*		Involved in stress response
*EPA1*	*C. glabrata*		Adhesin
*EPA3*	*C. glabrata*		Adhesin
*ERG1*	*Candida spp, Cryptococcus spp, Aspergillus spp*	ERG genes	Ergosterol biosynthesis pathway
*ERG10*	*Candida spp, Cryptococcus spp, Aspergillus spp*	ERG genes	Ergosterol biosynthesis pathway
*ERG11 (formerly ERG16)* *Cyp51A, Cyp51B*	*Candida spp*, *C. neoformans* *A. fumigatus*	ERG genes	Ergosterol biosynthesis pathway
*ERG13*	*Candida spp, Cryptococcus spp, Aspergillus spp*	ERG genes	Ergosterol biosynthesis pathway
*ERG2*	*Candida spp, Cryptococcus spp, Aspergillus spp*	ERG genes	Ergosterol biosynthesis pathway
*ERG24*	*Candida spp, Cryptococcus spp, Aspergillus spp*	ERG genes	Ergosterol biosynthesis pathway
*ERG25*	*Candida spp, Cryptococcus spp, Aspergillus spp*	ERG genes	Ergosterol biosynthesis pathway
*ERG27*	*Candida spp, Cryptococcus spp, Aspergillus spp*	ERG genes	Ergosterol biosynthesis pathway
*ERG3*	*Candida spp, Cryptococcus spp, Aspergillus spp*	ERG genes	Ergosterol biosynthesis pathway
*ERG4*	*Candida spp, Cryptococcus spp, Aspergillus spp*	ERG genes	Ergosterol biosynthesis pathway
*ERG5*	*Candida spp, Cryptococcus spp, Aspergillus spp*	ERG genes	Ergosterol biosynthesis pathway
*ERG6*	*Candida spp, Cryptococcus spp, Aspergillus spp*	ERG genes	Ergosterol biosynthesis pathway
*ERG7*	*Candida spp, Cryptococcus spp, Aspergillus spp*	ERG genes	Ergosterol biosynthesis pathway
*ERG9*	*Candida spp, Cryptococcus spp, Aspergillus spp*	ERG genes	Ergosterol biosynthesis pathway
*ERO1*	*C. albicans*		Involved in stress response
*FRE3*	*C. neoformans*		Involved in iron acquisition
*GRE99*	*C. albicans*		Involved in stress response
*GRP2*	*C. parapsilosis*		Involved in stress response
*HapE*	*A. fumigatus*	Transcription factors	CCAAT–binding transcription factor complex subunit, repressor of *cyp51A*
*HAPX*	*C. neoformans*	Transcription factors	Regulates iron acquisition and metabolism
*HMG1*	*Candida spp, Cryptococcus spp, Aspergillus spp*		Ergosterol biosynthesis pathway
*HSP70*	*C. albicans*		Involved in stress response
*HSP90*	*C. tropicalis*		Involved in stress response
*HYR1 (GPX1)*	*C. albicans*		Involved in stress response
*IFD5*	*C. albicans*		Putative aryl–alcohol dehydrogenase
*IPF5987*	*C. albicans*		Unknown function
*KRE6*	*C. albicans*		Involved in membrane and cell wall integrity
*MCM2*	*C. neoformans*		Member of the minichromosome maintenance (MCM) protein family
*MDR1 (formerly BEN^r^)*	*Candida spp*	MFS transporters	MFS transporter
*MDR2*	*A. fumigatus*	MFS transporters	MFS transporter
*MDR3*	*A. fumigatus*	MFS transporters	MFS transporter
*MDR4*	*A. fumigatus*	MFS transporters	MFS transporter
*mdrA*	*A. fumigatus*	MFS transporters	MFS transporter
*mfsC*	*A. fumigatus*	MFS transporters	MFS transporter
*MfsD*	*A. fumigatus*	MFS transporters	MFS transporter
*MKC1*	*C. tropicalis*		Involved in stress response
*MLH3*	*C. neoformans*		Endonuclease involved in DNA mismatch repair
*MNN23*	*C. albicans*		Involved in membrane and cell wall integrity
*MRR1*	*Candida spp*	Transcription factors	Activator of *MDR1*
*NCP1*	*C. albicans*		Involved in membrane and cell wall integrity
*NDT80*	*C. parapsilosis*	Transcription factors	Activator of *CDR1*
*PBS2*	*C. albicans*		Involved in stress response
*PDR1*	*C. glabrata*	Transcription factors	Activator of *SNQ2*, *CDR1* and *CDR2*
*PDR16*	*C. albicans*		Involved in membrane and cell wall integrity
*PMC1*	*C. neoformans*		Calcium transporter
*PtaB*	*A. fumigatus*		Regulates biofilm formation and conidiation
*RHB1*	*C. albicans*		Involved in membrane and cell wall integrity
*RPD3*	*C. neoformans*		Histone deacetylase
*RTA3*	*C. albicans*		*RTA1* like family protein
*RttA*	*A. fumigatus*		Putative protein responsible for tebuconazole tolerance
*SET101*	*C. neoformans*		Histone–lysine N–methyltransferase, H3 lysine–4 specific
*SNQ2*	*Candida spp, Cryptococcus spp, Aspergillus spp*	ABC transporters	ABC transporter
*Ssc70*	*A. fumigatus*	Chaperones	Involved in stress response
*TAC1*	*Candida spp*	Transcription factors	Activator of *CDR1*, *CDR2* and *PDR16*
*TPK1*	*C. albicans*		Involved in stress response
*UbcD*	*A. fumigatus*		Ubiquitin–conjugating enzyme E2
*UFD4*	*C. neoformans*		E3 ubiquitin–protein ligase
*UPC2*	*Candida spp, Cryptococcus spp, Aspergillus spp*	Transcription factors	Activator of *ERG11*
*YNL229C*	*C. albicans*		Involved in stress response
*YPL88*	*C. albicans*		Involved in membrane and cell wall integrity
*YPR127W*	*C. albicans*		Similar to aryl alcohol dehydrogenase
*YPX98*	*C. albicans*		Involved in membrane and cell wall integrity

Triazole resistance is an increasing problem worldwide (Sanglard, 2016). *Candida* spp. cause human infection ranging from systemic life-threatening to mucosal diseases (Whaley et al., 2017). Currently known triazole-resistance mechanisms in *Candida* spp. include mutations in the *ERG11* gene, which can be either homozygous or heterozygous, or overexpression *via* its transcriptional activator, Upc2 (Whaley et al., 2017; Ben-Ami and Kontoyiannis, 2021). Other mechanisms include inactivation of *ERG3*, encoding sterol C5,6-desaturase, leading to the incorporation of alternative sterols into the cell membrane, overexpression of efflux ATP-binding cassette (ABC) transporters due to mutations in the transcriptional activators *TAC1* (activating CDR1 and CDR2) and *MRR1* (activating MDR1, formerly known as BEN^r^), and loss of heterozygosity (LOH) and aneuploidy of key genes or chromosomes, such as chromosome 5 on which *ERG11* and *TAC1* are encoded (Whaley et al., 2017; Ben-Ami and Kontoyiannis, 2021). Amplification or deletion of a chromosomal segment is defined as copy number variation (CNV).

*Cryptococcus* spp. (mainly *C. neoformans* and *Cryptococcus gattii*) are encapsulated yeast that can cause life-threatening infections, primarily in immunocompromised patients (Zafar et al., 2019). While not many *ERG11* mutations have been identified in resistant strains, *C. neoformans* has a known transient resistance and hetero-resistance mechanism mediated by aneuploidy of key chromosomes (primarily chromosome 1 that encodes *ERG11* and the efflux transporter AFR1), that can result in upregulation of *ERG11* or *AFR1* (Zafar et al., 2019; Ben-Ami and Kontoyiannis, 2021).

*A. fumigatus* is an environmental filamentous fungus and the most common mold pathogen in humans. It can cause a wide range of diseases in humans, including invasive infections with high mortality rates in immunocompromised patients (Latgé and Chamilos, 2019; Nywening et al., 2020). The majority of resistance cases in *A. fumigatus* are due to mutations or overexpression of *ERG11/cyp51A* genes (Sanglard, 2016; Nywening et al., 2020). While single point mutations can elevate resistance, a tandem repeat (TR) of the SrbA transcriptional activator binding site, combined with a specific set of mutations in the reading frame, results in overexpression of the *cyp51A* gene and therefore elevates resistance. Additional triazole-resistance associated mechanisms include, among others, mutations in *hmg1* encoding HMG-CoA reductase (one of the first steps in the ergosterol biosynthesis pathway), mutations in *hapE* encoding a subunit of CCAAT-binding transcription factor complex (CBC) and overexpression of multidrug efflux transporters (Sanglard, 2016; Nywening et al., 2020).

**Figure 1:**
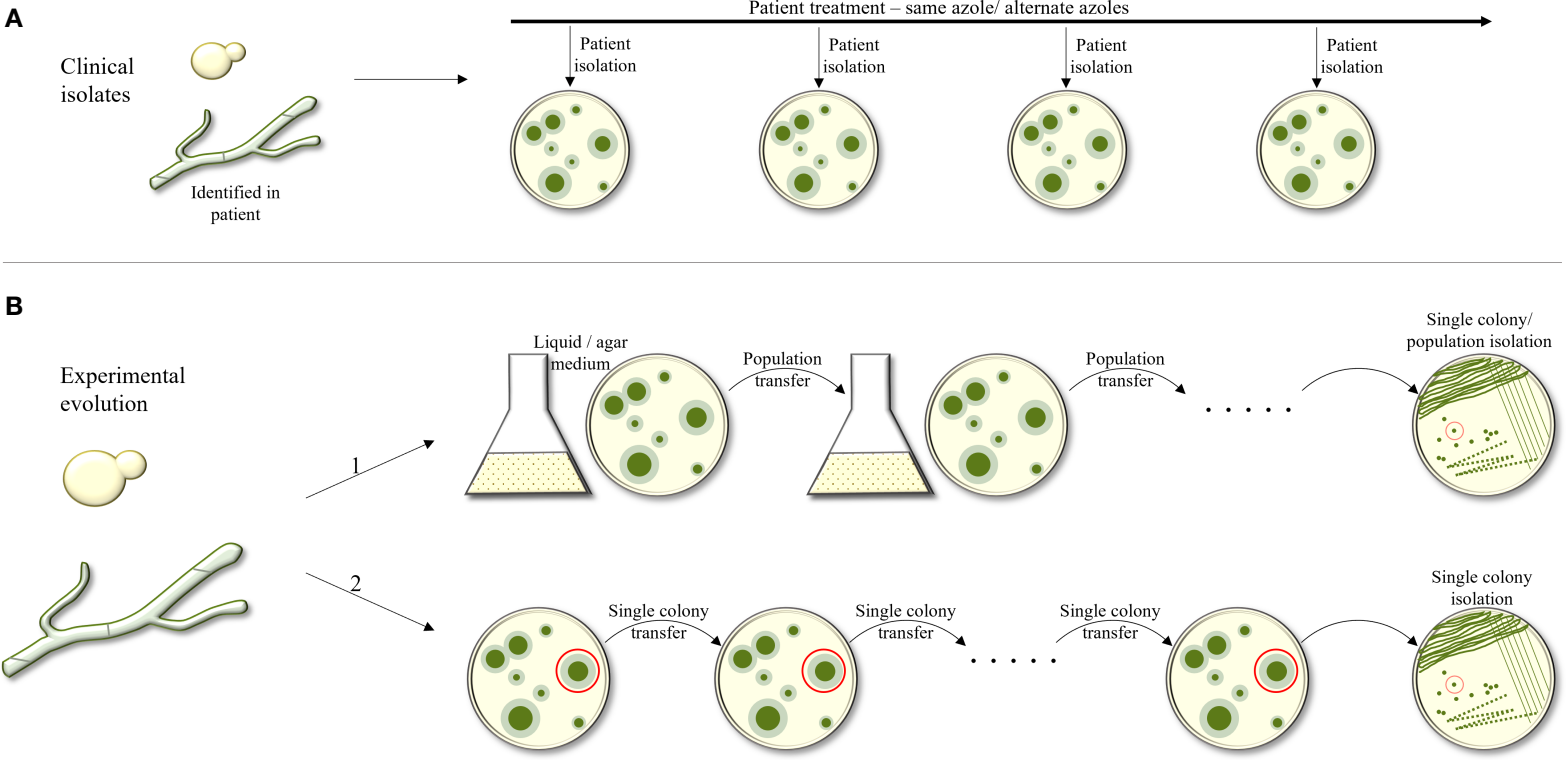
Outline of experimental approaches used to study evolution of triazole resistance. **(A)** Collection of serial fungal isolates from an infected patient over time. Isogenic strains are identified and compared. **(B)** Experimental evolution *in–vitro*. The fungus is passaged under increasing tiazole concentrations, in liquid broth or on agar plates. In B1, a population of the strain under selection is collected and passaged onto the next agar plate or broth tube, containing increasing antifungal concentrations. In B2, during each passage, only one colony or CFU is selected from the agar plate and transferred to the next plate.

Evolutionary experiments are an important tool to study the processes occurring within a fungus in response to different stressors. There are two categories of evolutionary experiments: collection of serial fungal isolates from infected triazole-treated patients, and *in-vitro* experiments in which a fungus is passaged under increasing triazole concentrations, either in liquid broth or on agar plates (**Figure 1**). The advantage of collecting serial clinical isolates is the possibility of monitoring real-life changes within the host during drug treatment. The disadvantage is the lack of identical growth conditions of the isolates and having many non-triazole-related differences between them as they are not always isogenic. The advantage of *in-vitro* evolution is the ability to control every aspect of the experiment, using all-isogenic strains and monitoring every generation. The clear disadvantage is the lack of an actual physiological environment.

*In-vitro* evolution experiments can again be divided into two categories: in the first, a population of a specific starting strain is collected and passaged to the next agar or broth plate with increasing or steady antifungal concentration. In the second, in each passage, only one colony forming unit (CFU) is picked from one agar plate and transferred to the next. The advantage in the first approach is that it allows competition among the mutating colonies, favoring the most fitness-effective mutations that allow antifungal resistance. In the second, since only one colony is passaged, there is no selection against antifungal-resistance mutations that also have a fitness cost.

Regardless of the approach used to perform *in-vitro* evolution, a control lineage must be passaged in triazole-free media, in parallel to the evolving triazole-treated lineages. Based on our experience, the evolved and control strains can differ by several hundred SNPs by WGS, and most are probably unrelated to triazole resistance. Therefore, multiple triazole-resistant lineages are evolved, and only SNPs occurring in several of the lineages independently are further analyzed by reintroduction into a susceptible laboratory strain.

This review will describe the *in-vitro* evolution experiments and serial clinical isolate studies performed in pathogenic fungi during triazole exposure. We will outline the identified resistance mechanisms and compare the findings reported using the various experimental approaches, while focusing more closely on recent publications.

### Candida species

Patients infected with *Candida* spp. are treated primarily with fluconazole, and fungal resistance can develop over time. The following section will describe a few studies performed with serial clinical isolates, and by *in-vitro* evolution, and discuss the commonalities and differences between species and experiments. The number of studies using serial clinical isolates of C*andida albicans* is impressive. Therefore, the citations and examples described here should be considered representative rather than comprehensive.

#### Serial clinical isolates outline the timeline of genetic changes occurring in *Candida* spp. during the acquisition of triazole resistance

In this approach, strains were isolated from patients during treatment and tested for drug susceptibility and isogeneity. Several experimental approaches were used to study the isolated strains, including, in earlier studies, sequencing of specific genes for the detection of SNPs, and Southern and Northern blot analysis of specific genes to detect sequence and expression variations. In later studies, omics approaches, including RNAseq, proteomics and whole-genome sequencing (WGS) were used to identify SNPs, expression changes, and gene copy number variations in the entire genome that may be associated with triazole resistance. The subsequent introduction of resistance-associated alleles into susceptible yeasts is a valuable tool for determining a mutation’s importance in the acquisition of triazole resistance. In *C. albicans* (**Table 2**), most serial clinical isolate studies support the connection between increased expression of *CDR1* and *CDR2*, encoding efflux ABC transporters, and *MDR1*, encoding an efflux transporter from the major facilitator superfamily (MFS), to fluconazole resistance (Sanglard et al., 1995; White, 1997; Sanglard et al., 1998; Perea et al., 2001; Rogers and Barker, 2003; Coste et al., 2006; Coste et al., 2007; Dunkel et al., 2008; Ford et al., 2015; Bhattacharya et al., 2016; Song et al., 2022). In some cases, this was linked to hyperactive alleles of their transcriptional activators, TAC1 (Coste et al., 2007; Ford et al., 2015) and MRR1 (Dunkel et al., 2008; Ford et al., 2015), respectively. *ERG11* triazole-resistance-associated substitutions, either homozygous or heterozygous, were found in several clinical isolates sets (White, 1997; Sanglard et al., 1998; Perea et al., 2001; Coste et al., 2006; Coste et al., 2007; Ford et al., 2015). Several studies also described elevated expression of *ERG11* in resistant isolates (White, 1997; Perea et al., 2001; Coste et al., 2007; Heilmann et al., 2010; Hoot et al., 2011; Bhattacharya et al., 2016; Latgé and Chamilos, 2019), compared to their isogenic susceptible strains, that in some cases can be linked to mutations or overexpression in their transcriptional activator UPC2 (Heilmann et al., 2010; Hoot et al., 2011). Loss of heterozygosity (LOH) can also contribute to triazole resistance by enabling mutated resistance-associated alleles, such as *TAC1*, *ERG11*, or areas on chromosome 3 (on which *CDR1*, *CDR2* and *MRR1* are encoded), to duplicate and exert a stronger effect (White, 1997; Coste et al., 2007; Dunkel et al., 2008; Selmecki et al., 2008; Ford et al., 2015). Aneuploidy and isochromosome formation increases the copy number of the genes located in the duplication area, such as chromosome 5 (on which *ERG11* and *TAC1* are encoded, or isochromosome forming i(5L)) (Coste et al., 2007, Selmecki et al., 2008), which results in elevated expression. These main resistance mechanisms are repeatedly evolved in clinical strains isolated from different infection sites. Less common findings are overexpression of several *ERG* genes, including *ERG1*, *ERG2*, *ERG3*, *ERG5*, *ERG6*, *ERG9*, and *ERG10* (Rogers and Barker, 2003; Hoot et al., 2011).

**Table 2:** Development of triazole resistance in serial clinical isolates of *Candida* spp.

Species	Isolate source	Approach used	Main findings	Reference
*C. albicans*	Serial clinical isolates from AIDS patients with oropharyngeal candidiasis	Accumulation of [^3^H] fluconazole, DNA probe hybridizations, Northern blot quantification of *ERG11*, *CDR1* and *MDR1.* Sequencing of *ERG11*, testing mutation importance by transformation of susceptible *Ca*.	Increase in *CDR1, MDR1* and *ERG11* expression, lower accumulation of intracellular fluconazole. Erg11 mutations linked to triazole resistance: Y132H, S405F, G464S and R467K, due to change in the affinity of Erg11 to the triazole.	(Sanglard et al., 1995), (Sanglard et al., 1998)
*C. albicans*	Serial clinical isolates from AIDS patients with oropharyngeal candidiasis, (Pfaller et al., 1994)	Northern blot quantification of *ERG11*, *MDR1*, *CDR1* and more.	Increase in *ERG11* expression (referred to as *ERG16*), in addition to point mutation and LOH in *ERG11*, increase in *MDR1* and *CDR1* expression.	(White, 1997)
*C. albicans*	Serial clinical isolates from AIDS patients with recurrent episodes of oral thrush	Southern hybridization, Northern hybridization, sequencing of *CDR4*.	CDR4 expression is not enhanced in fluconazole–resistant *C. albicans* isolates, inactivation of CDR4 does not increase fluconazole susceptibility.	(Franz et al., 1998)
*C. albicans*	Serial clinical isolates from AIDS patients with oropharyngeal candidiasis	Northern blot quantification of *CDR1* and *CDR2, MDR1* and *ERG11*, sequencing of *ERG11.*	Increase in *CDR1* and *CDR2, MDR1* and *ERG11* expression. Erg11 substitutions linked to triazole resistance: D116E, G450E, G307S, Y132F, D446N, G464S, F126L, K143R, S405F, F449S, and T229A.	(Perea et al., 2001)
*C. albicans*	Serial clinical isolates from AIDS patients with oropharyngeal candidiasis [Isolates taken from (White, 1997) and (Redding et al., 1994)]	Microarray, RT–PCR.	Increase in *CDR1* and *CDR2, MDR1, ERG2, GPX1*, *RTA3*, *IFD5*, *IPF5987*, and *CRD2* expression.	(Rogers and Barker, 2003)
*C. albicans*	Serial clinical isolates from AIDS patients with oropharyngeal candidiasis [Isolates taken from (Sanglard et al., 1995)]	Southern blots, Northern blots, Immunoblots, RT–PCR, gene sequencing efflux activity fluorescent assays, gene disruption, testing mutation importance by transformation of susceptible *Ca*.	CDR1 and CDR2 levels are constitutively high in triazole–resistant isolates due to hyperactive alleles of the TAC1 transcriptional activator: V736A, G980E, and deletions ΔM677 and Δ962–969. LOH on chromosome 5. *ERG11* mutations linked to triazole resistance, *ERG11* elevated expression, i(5L) formation.	(Coste et al., 2006), (Coste et al., 2007)
*C. albicans*	Serial clinical isolates from bone marrow transplant (BMT) patient [collected from rectal, blood and lung tissue isolates, (Marr et al., 1997)]	Southern analysis for Chr5L probe, restriction enzyme digestion to differentiate between genes’ alleles, testing genes’ importance by transformation–deletion.	LOH in *TAC1*, i(5L) formation, chromosome 5 breakage in one isolate, increased *TAC1* and *ERG11* copy number on Chr5L.	(Selmecki et al., 2008)
*C. albicans*	Serial clinical isolates from several sources	Gene sequencing, FACS analysis, testing mutation importance by transformation of susceptible *Ca*.	MRR1 substitutions linked to triazole resistance: K335N, Q350L, T360I, P683H, N803D, G878E and T896I. MDR1 overexpression due to MRR1 substitutions. LOH caused by mitotic recombination and chromosome loss.	(Dunkel et al., 2008)
*C. albicans*	Serial clinical isolates from bone marrow transplant (BMT) patient [collected from rectal, blood and lung tissue isolates, (Selmecki et al., 2008)]	*UPC2* and *ERG11* sequencing and qRT–PCR, testing mutation importance by transformation of susceptible *Ca*.	Increase in *UPC2* and *ERG11* expression, UPC2 A643T gain–of–function mutation that results in *ERG11* overexpression.	(Heilmann et al., 2010)
*C. albicans*	Serial clinical isolates from AIDS patients with oropharyngeal candidiasis [Isolates taken from (White, 1997)]	*UPC2* sequencing, ergosterol quantitation, testing mutation importance by transformation of susceptible *Ca*, and qRT–PCR.	UPC2 A643T gain–of–function mutation that results in *ERG1, ERG2, ERG3, ERG5, ERG6, ERG9, ERG10* and *ERG11* overexpression, and increased ergosterol levels.	(Hoot et al., 2011)
*C. albicans*	Serial clinical isolates from AIDS patients with oropharyngeal candidiasis [Isolates taken from (White, 1997) and (Perea et al., 2001)]	Whole genome sequencing, ploidy analysis by flow cytometry, gene ontology (GO) and functional enrichment.	LOH on chromosome 5 as well as on chromosome 3, on which *CDR1* and *CDR2* and *MRR1* are encoded, genetic diversity within sequential isolates, mutations in *ERG11*, *TAC1, MRR1* and many more including fungal–type cell wall and surface genes. changes in *in–vitro* fitness, filamentation, adhesion, and *in–vivo* virulence.	(Ford et al., 2015)
*C. albicans*	Serial clinical isolates from patients with *Candida* vaginitis	Efflux activity fluorescent assays, qRT–PCR for *CDR1*, *CDR2*, *MDR1*, and *ERG11* expression levels.	Increase in *CDR1* and *CDR2, MDR1* and *ERG11* expression, increased efflux activity.	(Bhattacharya et al., 2016)
*C. albicans*	Serial clinical isolates from AIDS patients with oropharyngeal candidiasis (Isolates taken from, (White, 1997)	Proteomics analysis, bioinformatic analyses of gene ontology (GO) and subcellular functional annotations.	CDR1 upregulation, differentially expressed proteins are mainly involved in the membrane, cell, organelle, catalytic activity, binding, transporter activity, metabolic process, single–organism process, and cellular process.	(Song et al., 2022)
*C. glabrata*	Serial clinical isolates isolated from blood	qRT–PCR, sequencing of *ERG11.*	Increase in *CDR1* and *CDR2* and *SNQ2* (ABC transporter) expression.	(Sanguinetti et al., 2005)
*C. glabrata*	Serial clinical isolates from AIDS patients with OPC [Isolates taken from (Sanglard et al., 1999)]	Whole genome sequencing, RNAseq, gene sequencing, adhesins predictions.	Transcription factor *CgPDR1* gain–of–function mutation, can modulate adhesin EPA1 expression and increase host cells adherence. Chromosomal rearrangements. Presence of transposon–like genes and adhesin–like genes.	(Vale-Silva et al., 2017)
*C. glabrata*	Serial clinical isolates isolated from sputum, stools, or midstream urine	Gene sequencing, disruption, reinstitution of *CgPDR1*, adherence assays, qRT–PCR.	*CgPDR1* gain–of–function mutations induce efflux transporters expression, triazole resistance, and host cells adherence due to different adhesin expression profiles.	(Ni et al., 2018)
*C. krusei*	Serial clinical isolates isolated from a single patient’s blood, stools, and then bronchial secretions	qRT–PCR for *ABC1*, *ABC2*, and *ERG11*, sequencing of *ERG11*.	Increase in *ABC1* (ABC transporter) expression, increased *ERG11* expression in one isolate, Erg11 substitutions linked to triazole resistance: Y140H.	(Ricardo et al., 2014)
*C. parapsilosis*	Serial clinical isolates a patient’s blood culture	Sequencing of *ERG11*, *MRR1*, *TAC1* and *UPC2*. qRT–PCR.	L986P mutation in MRR1, increased expression of *MRR1* and *MDR1.*	(Zhang et al., 2015)
*C. auris*	Serial clinical isolates from a single patient isolated from several sources	Whole genome sequencing, examination of *ERG11*, *MDR1*, *CDR1*, *FKS1*, *ERG2*, *ERG3*, *ERG5* and *ERG6*.	Erg11 substitutions linked to triazole resistance: V125A and F126L.	(Biswas et al., 2020)

**Figure 2:**
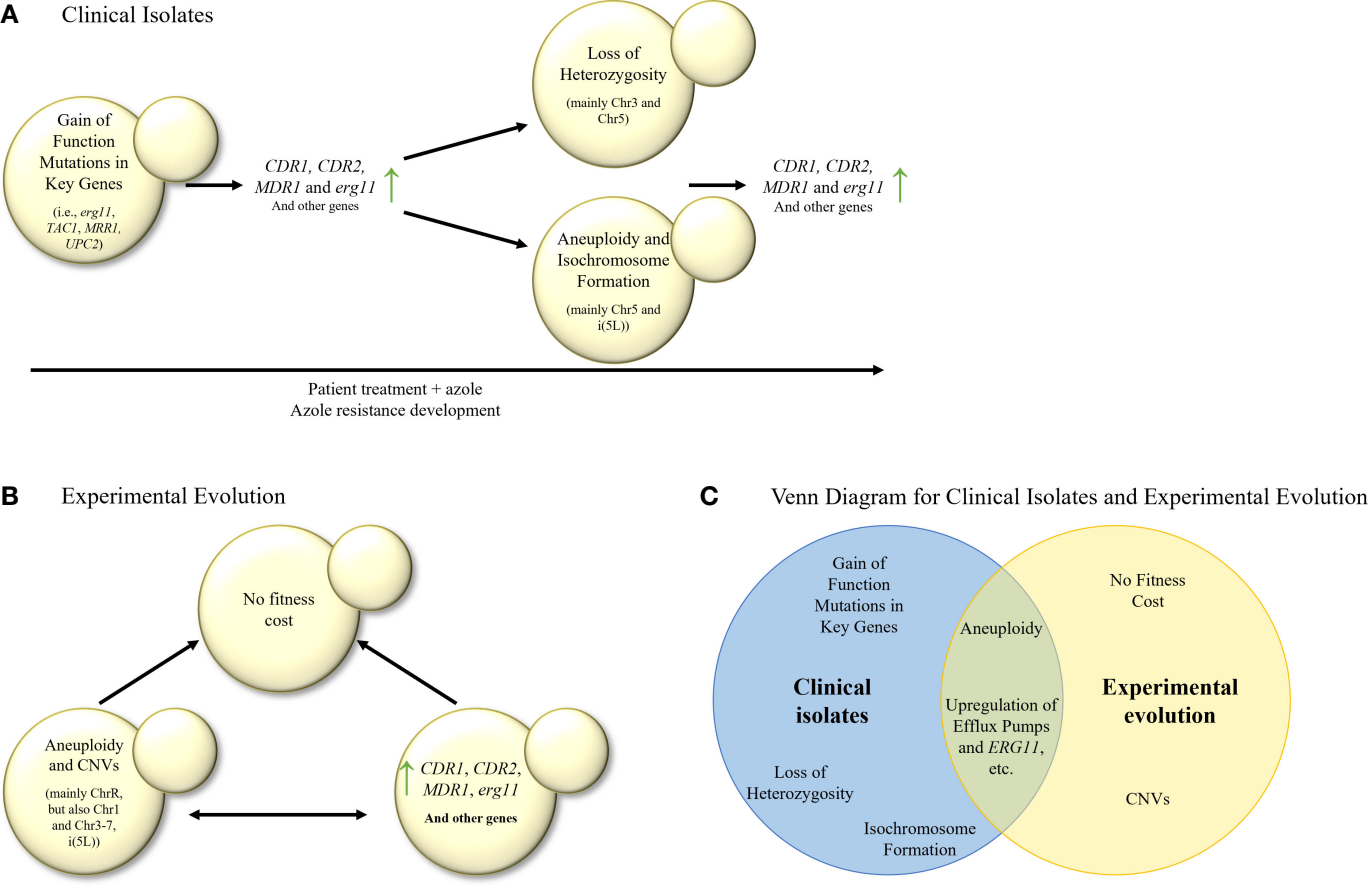
**(A)** Stepwise evolution of triazole resistance in serial clinical isolates of *C. albicans*. Gain–of–function mutations are followed by elevation in the expression of efflux transporters and ergosterol biosynthesis *(erg)* genes, then by LOH, aneuploidy and isochromosome formation, and finally by another increase in expression of efflux transporters and *erg* genes, leading to triazole resistance. **(B)** *In–vitro* evolution of triazole resistance in *C. albicans* isolates. Elevation in the expression of efflux transporters, *erg* genes and many other genes is commonly observed, as well as aneuploidies and CNVs in most of the chromosomes. It is unknown whether overexpression of key genes precedes aneuploidies and CNVs, or vice versa. Usually, no fitness cost was found in the evolved isolates. **(C)** Venn diagram of azole resistance mechanisms in serial clinical isolates and in *in–vitro* evolution in *C. albicans* isolates. Aneuploidy and upregulation of efflux pumps and *ERG11* are common to both evolutionary tracks.

There seems to be agreement that the first step of triazole-resistance evolution in serial clinical isolate studies in *C. albicans* involves the acquisition of gain-of-function/hyperactive/resistance-associated mutations or alleles in triazole-resistance associated genes, such as *ERG11*, *TAC1* or *MRR1*, often accompanied by *CDR1*, *CDR2* and *MDR1* overexpression (Sanglard et al., 1995; White, 1997; Sanglard et al., 1998; Perea et al., 2001; Rogers and Barker, 2003; Coste et al., 2006; Dunkel et al., 2008; Ford et al., 2015; Bhattacharya et al., 2016; Latgé and Chamilos, 2019; Song et al., 2022) (**Figure 2A**). The next steps include LOH of key genes (for example, *ERG11*) or chromosome areas (for example, chromosome 3 or chromosome 5), aneuploidy of chromosome 5 and i(5L), although there are conflicting results on what event precedes the others. Some researchers hypothesized that aneuploidy of chromosomes such as i(5L) can promote LOH (Ford et al., 2015).

In *Candida glabrata*, serial clinical isolate analysis identified a gain-of-function mutation in the transcription factor CgPDR1 (Vale-Silva et al., 2017; Ni et al., 2018), associated with increased expression of efflux transporters *CDR1*, *CDR2*, and *SNQ2* (Sanguinetti et al., 2005; Ni et al., 2018) (**Table 2**). Similarly, a gain–of–function mutation in the transcription factor MRR1 was described in *Candida parapsilosis* and was linked to increased expression of both *MRR1* and *MDR1* (Zhang et al., 2015). In *Candida krusei*, *ABC1* overexpression was described as well, along with *ERG11* point mutation and increased *ERG11* expression in one isolate (Ricardo et al., 2014), while in *Candida auris* only *ERG11* point mutations were found (Biswas et al., 2020).

When comparing *C. albicans* to other *Candida* species, serial clinical isolate studies suggest that while *ERG11* mutation or overexpression is a common triazole–resistance mechanism in *C. albicans*, *C. auris* and *C. krusei*, in other *Candida* species such as *C. glabrata* and *C. parapsilosis*, expression of efflux transporters is mainly seen, resulting from gain–of–function mutations in their respective transcription factors. Although *ERG11* mutations have been previously described in clinical isolates of *C. glabrata* (Silva et al., 2016) and *C. parapsilosis* (Whaley et al., 2017), none were found in the serial clinical isolates described here. In addition, unlike other *Candida* species, in *C. glabrata, ERG11* alterations are not a major resistance mechanism (Whaley et al., 2017; Ben-Ami and Kontoyiannis, 2021).

#### *Candida* spp. *in–vitro* evolution involves elevation in expression of efflux transporters, *ERG* genes as well as aneuploidies and CNVs

In this approach, a small number of strains, often a single strain, are serially passaged in broth containing either constant or increasing triazole concentrations. The last generation is either analyzed as a population or isolated to a single CFU that is studied. All generations are stored and can be analyzed for the timing of individual mutations during the evolutionary process. At the same time, a control group is usually transferred for the same number of generations without triazole. Several experimental approaches are used to study the strains or populations. They include, in earlier studies, checking for ploidy changes, testing of resistance stability as a sign for an underlying genetic mechanism, sequencing of specific genes for the detection of SNPs, and Southern and Northern blot analysis of specific genes. Later studies use WGS and RNAseq to identify SNPs, changes in expression and gene copy number, that may be associated with triazole resistance. The final stage is the verification of mutations by reintroduction, alone and in combination, into a susceptible strain to assess their relative contributions to resistance.

In *C. albicans*, most *in–vitro* evolution studies, similarly to clinical isolate studies, describe an increased expression of *ERG11*, *CDR1*, *CDR2* (Cowen et al., 2000), and *MDR1* (Cowen et al., 2000; Dunkel et al., 2008). Increased expression of *MDR1* can be partially explained by transcription factor *MRR1* gain–of–function mutations and LOH (Dunkel et al., 2008) (**Table 3**; **Figure 2B**). Increased expression of *CDR1* and *CDR2* may be explained by mutations in transcription factor TAC1, or other unknown factors. In addition, changes in expression (either induction or repression of expression) were observed in *ERG1*, *ERG3*, and *ERG13* (Silva et al., 2016). ChrR (chromosome R, encoding rDNA genes) changes have also been frequently described (Cowen et al., 2000; Huang et al., 2011; Kukurudz et al., 2022), along with Chr3 (containing *CDR1* and *CDR2*), as well as Chr4 and Chr6 aneuploidies, where the resistance mechanism remains unclear (Huang et al., 2011; Todd et al., 2019; Todd and Selmecki, 2020; Kukurudz et al., 2022). Overexpression and CNVs of genes involved in membrane and cell wall integrity (*YPL88*, *YPX98*, *PDR16*, *CRZ1*, *CDR3*, *NCP1*, *ECM21*, *MNN23*, *RHB1*, and *KRE6*), stress response (*HYR1*, *GRE99*, *YNL229C*, *HSP70*, *CGR1*, *ERO1*, *TPK1*, *ASR1*, and *PBS2*) have also been reported, none of which have been proven to cause triazole resistance (**Table 3**) (Cowen et al., 2002; Todd and Selmecki, 2020). A high copy number of chromosomal segments, including i(5L) containing *ERG11*, was described, but not recurrently (Todd and Selmecki, 2020). Despite all this data, it has yet to be shown which processes occur at the beginning of the experimental evolution and support later adaptations (**Figure 2B**). No ERG11 mutations were described in any of the described studies.

**Table 3:** Development of triazole resistance in *Candida* spp. isolates undergoing *in–vitro* evolution.

Species	Isolate source	Evolution method	Total number of passages	Approach used	Main findings	Reference
*C. albicans*	ATCC 36082 – originally a clinical isolate	Passages in four constant concentrations of **fluconazole** in broth.	4–10	Northern blot for *ERG11*, *CDR1* and *MDR1* expression, karyotype analysis, mice model fungal burden analysis.	Speed and extent of fluconazole resistance acquisition depends on the drug concentration used during evolution	(Calvet et al., 1997)
*C. albicans*	Clinical isolate from an HIV positive patient	Stepwise increases of **fluconazole** in broth	330	DNA probe hybridizations, Northern and Southern blots, sequencing of *ERG11*, heterozygosity testing, CHEF karyotype analysis, fitness tests, microarray.	Different patterns of overexpression of *ERG11*, *CDR1*, *CDR2* and *MDR1* in different strains. Size variation in ChrR in both resistant and control strains. No fitness cost in most final resistant populations. Overexpression of *YPL88*, *YPX98*, *YPR127W*, *ADH4*, *HYR1*, *GRE99*, *YNL229C* and *PDR16*. Different patterns of decreased or increased expression of *ERG1*, *ERG3* and *ERG13* in different isolates.	(Cowen et al., 2000), (Cowen et al., 2001), (Cowen et al., 2002)
*C. albicans*	Laboratory strains from (Riggle and Kumamoto, 2006)	Stepwise increases of **fluconazole** in broth	11	Southern blots, sequencing of *MRR1*, *CAP1*, *MEP1*, *SAP9* and *AAP1*, genetic manipulation of *MRR1*.	MRR1 substitutions linked to triazole resistance in resistant strains constitutively overexpressing *MDR1*: T381I, R873T, A880E, W893R and L998F. LOH in MRR1 is caused by mitotic recombination and chromosome loss.	(Dunkel et al., 2008)
*C. albicans*	SC5314 – originally a clinical isolate	Stepwise increases of **fluconazole** in broth of three fluorescently labeled populations.	172	CHEF karyotype analysis, ploidy analysis by flow cytometry, microarray, fitness cost of drug resistance.	Tetraploidy and triploidy detected in one control isolate and one evolved isolate, respectively. No i(5L) formation detected, size variation in ChrR. No fitness cost associated with increased drug resistance.	(Huang et al., 2011)
*C. albicans*	Clinical isolates	Passages in a constant concentration of **fluconazole** in broth or **miconazole** agar plates.	10	CHEF karyotype analysis, Southern blot, flow cytometry, WGS.	i(4R) formation (Chr4R isochromosome) with no fitness cost, segmental aneuploidies.	(Todd et al., 2019)
*C. albicans*	SC5314, and three other clinical isolates	Passages in a constant concentration of **fluconazole** in broth.	10	CHEF karyotype analysis, Southern blot, gene ontology (GO) analysis, WGS, copy number variation (CNV) analysis.	Novel CNVs and high copy number in Chr1R, Chr3R, Chr4L, Chr5L, aneuploidy in Chr7R and i(5L). Novel CNVs are flanked by distinct long inverted repeat sequences and increase fitness and tolerance to at least one triazole. Amplified copy number of *MRR1*, *CDR1*, *CDR2*, *CRZ1*, *CDR3*, *NCP1*, *ECM21*, *MNN23*, *RHB1*, *KRE6*, *HSP70*, *CGR1*, *ERO1*, *TPK1*, *ASR1*, and *PBS2*.	(Todd and Selmecki, 2020)
*C. albicans*	Clinical isolates	Passages in a constant concentration of **fluconazole** in broth.	10	OD measurements, flow cytometry.	Adaptation is influenced by strain background, increase in median genome size.	(Gerstein and Berman, 2020)
*C. albicans*	Clinical isolates	Passages in a constant concentration of **posaconazole** in broth.	4	Ploidy analysis, WGS, karyotype analysis.	Inconsistent tradeoff between increased fitness in the presence and in the absence of triazole, higher increase in tolerance to posaconazole, rather than resistance, cross–tolerance to other triazoles. ChrR trisomy, Chr3, Chr4 and Chr6 aneuploidies.	(Kukurudz et al., 2022)
*C. parapsilosis*	Clinical isolate	Passages in a constant concentration of **fluconazole**, **voriconazole**, or **posaconazole** in broth.	60	Microarray, gene ontology (GO) analysis, qRT–PCR, sequencing of *MRR1*, *UPC2*, and *NDT80.*	Similar resistance profiles for fluconazole and voriconazole evolved strains, in those: overexpression of *MDR1* and two other MFS– family members, *PDR16*, *MRR1*, *GRP2*, several putative aldo–keto reductases and NADPH oxidoreductases, decreased expression of *SNQ2*, *ERG1*, *ERG2*, *ERG3*, *ERG11* and *ERG25*. In the posaconazole evolved strain: overexpression of *NDT80*, *UPC2*, *CDR2, PDR16*, *HMG1*, *ERG2*, *ERG3*, *ERG4*, *ERG5*, *ERG6*, *ERG7*, *ERG9*, *ERG10*, *ERG11*, *ERG13*, *ERG24*, *ERG25* and *ERG27*. Changes in copper transport and iron mobilization–related genes. MRR1 G583R and K873N substitutions in the fluconazole and voriconazole evolved strains, respectively.	(Silva et al., 2011)
*C. krusei*	Clinical isolate from a leukemia patient	Passages in a constant concentration of **voriconazole** in broth.	30	qRT–PCR for *ABC1*, *ABC2*, and *ERG11*, sequencing of *ERG11*. Effects of the efflux blocker tacrolimus (FK506).	Overexpression of *ABC1* in 3/4 isolates and of *ERG11* in 2/4 isolates. No *ERG11* mutations. Reverse of susceptibility in the presence of FK506.	(Ricardo et al., 2014)
*C. glabrata*	Clinical isolate	Passages in a constant concentration of **fluconazole** in broth.	80	Microarray, disruption of several *C. glabrata* genes, qRT–PCR, ergosterol quantification, biofilm quantification, adherence assay.	Triazole cross–resistance in the evolved population: posaconazole at 21 days, clotrimazole at 31 days, fluconazole and voriconazole at 45 days of exposure to fluconazole. *ERG11* overexpression in 31 days, no change in ergosterol levels. *CDR1* and *CDR2* overexpression in 31 and 45 days, PDR1 Y372C gain–of–function mutation. Decreased intracellular triazole accumulation, increased adhesin–encoding genes (especially *CgEpa3*) and biofilm formation.	(Cavalheiro et al., 2018)
*C. tropicalis*	Clinical isolates	Stepwise increases of **fluconazole** in broth	90	qRT–PCR, sequencing of *ERG11, UPC2, ERG1* and *ERG3.*	Triazole cross–resistance in some of the evolved populations, overexpression of *CDR1*, *CDR2, CDR3, MDR1, ERG1, ERG2, ERG3* and *ERG11*, *TAC1* and *UPC2, HSP90* and *MKC1*.	(Paul et al., 2020)
*C. auris*	Clinical isolate	Passages in a constant or stepwise increases in concentrations of **fluconazole** in broth	30	WGS, allele–specific PCR,	FS191S deletion in TAC1b, segmental Chr1 duplication, *CDR1* and *ERG11* overexpression. Chr5 duplication in a caspofungin and fluconazole–resistant strain, *CDR2* and *TAC1b* overexpression.	(Carolus et al., 2021)

Compared with the clinical isolate studies described above, the laboratory evolutionary studies find less gain–of–function mutations in *TAC1* and *UPC2*, with less isochromosome formation and more aneuploidies of chromosomes other than Chr5 (Huang et al., 2011; Todd et al., 2019; Todd and Selmecki, 2020; Kukurudz et al., 2022). In addition, *in–vitro* evolution studies more frequently describe the overexpression or CNVs of genes outside the ergosterol–biosynthesis pathway (Silva et al., 2016; Todd and Selmecki, 2020). In contrast, both study approaches report the overexpression of *ERG11*, *CDR1*, *CDR2*, and *MDR1* (Sanglard et al., 1995, White, 1997; Perea et al., 2001; Rogers and Barker, 2003; Coste et al., 2006; Dunkel et al., 2008, Heilmann et al., 2010; Hoot et al., 2011; Bhattacharya et al., 2016; Song et al., 2022), and gain–of–function mutations in *MRR1* (Dunkel et al., 2008; Ford et al., 2015).

Interestingly, clinical isolate studies describe ERG11 mutations while *in–vitro* evolution studies do not. One hypothesis for this is that in clinical studies, isolates experience a hostile in–host environment, which requires any adaptations to be minimalist and with no fitness cost, such as a single point mutation yielding azole resistance. On the other hand, *in–vitro* evolution studies are conducted in a clean, constant environment, allowing more room for fitness–costing changes that might yield higher azole resistance.

In general, *Candida* spp., other than *C. albicans*, also show high involvement of efflux transporters overexpressed in laboratory–evolved triazole–resistant strains (**Table 3**). In *C. parapsilosis* under fluconazole or voriconazole selection, there is strong elevation in expression of efflux transporters and aldo–keto reductases and NADPH oxidoreductases, which may help protect the cells from oxidative stress caused by triazole treatment (Silva et al., 2011). In contrast, in posaconazole–evolved strains, the data suggests that increased production of ergosterol is the primary mechanism of triazole resistance, as many genes of the ergosterol pathway are overexpressed. In *C. krusei* (Ricardo et al., 2014), overexpression of the *ABC1* transporter and *ERG11* was observed. The addition of the efflux blocker tacrolimus (FK506) reversed the susceptibility of the evolved strains to voriconazole.

Surprisingly, and for unknown reasons, *C. glabrata* (Cavalheiro et al., 2018), that was exposed to fluconazole, first acquired posaconazole resistance, followed by resistance to clotrimazole and then fluconazole and voriconazole. A PDR1 transcription factor Y372C gain–of–function mutation was followed by *ERG11*, *CDR1* and *CDR2* overexpression. Strains showed decreased intracellular triazole accumulation, increased expression of adhesin–encoding genes (especially CgEpa3) and biofilm formation.

In *C. tropicalis* (Paul et al., 2020), overexpression of *CDR1, CDR2, CDR3, MDR1, ERG1, ERG2, ERG3, ERG11, TAC1, UPC2, HSP90* and *MKC1* was observed in the resistant isolates, along with triazole cross–resistance in some. In *C. auris* (Carolus et al., 2021), one study detected a TAC1b FS191S deletion in the fluconazole–resistant strain, as well as a Chr1 aneuploidy in a segment containing the *ERG11* gene, resulting in increased expression. In a *C. auris* strain that was exposed to both caspofungin and fluconazole, a Chr5 duplication was also observed, correlating with overexpression of *TAC1b* (which is encoded on Chr5) and *CDR2*, but not *CDR1* (Carolus et al., 2021).

### Cryptococcus species

Patients infected with *Cryptococcus* spp. are often treated with amphotericin B and flucytosine combination therapy followed by fluconazole monotherapy (Ben-Ami and Kontoyiannis, 2021), leading to the development of triazole resistance. Triazole resistance studies based on serial clinical isolates or lab evolution have focused on *Cryptococcus neoformans*, the major pathogen in this group. Few resistance mutations in *ERG11* have been documented in resistant clinical *C. neoformans* isolates. The main triazole resistance mechanisms found were mediated by aneuploidy of key chromosomes (primarily chromosome 1 that encodes *ERG11* and the efflux transporter *AFR1*), that in turn cause elevated expression of the genes encoded on them (Stone et al., 2019). These unstable genomic reorganizations lead to transient resistance and hetero–resistance, a state in which a resistant subpopulation exists within a largely susceptible population (Stone et al., 2019).

#### Analysis of *C. neoformans* serial clinical isolates reveals elaborate genomic changes during acquisition of fluconazole resistance

**Figure 3:**
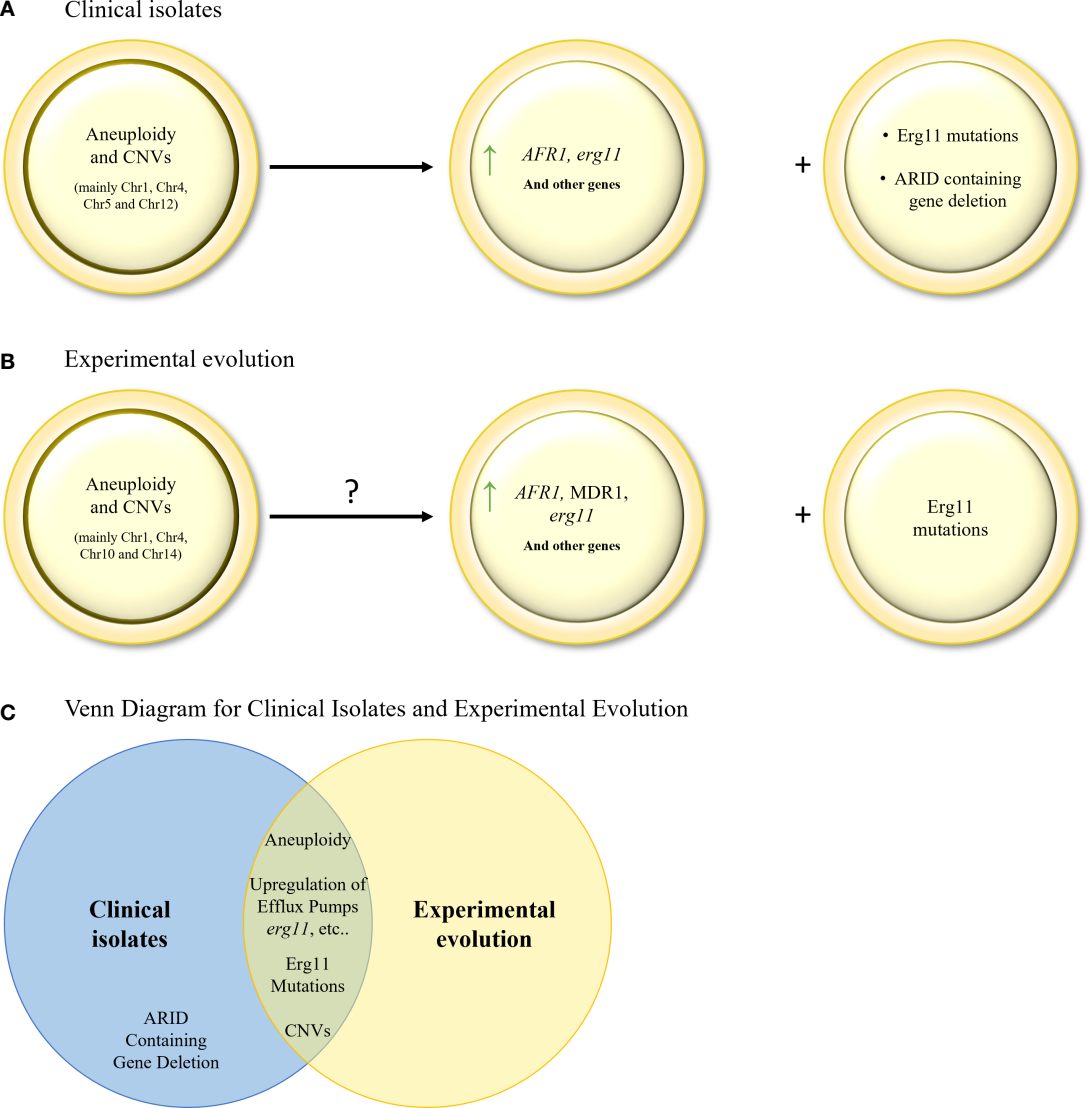
**(A)**. Evolution of triazole resistance in serial clinical isolates of *C neoformans*. Aneuploidies and CNVs in key chromosomes, leading to gene (*AFR1* and *ERG11*) overexpression, are the main drivers of triazole resistance in *C neoformans*. *ERG11* mutations and *ARID* containing gene deletion are also found, but to a lesser extent. **(B)** Mechanisms of triazole resistance identified in *in–vitro* evolution isolates of *C neoformans* and *C gattii*. Aneuploidies and CNVs in key chromosomes, leading to gene overexpression, have been identified. *ERG11* mutations were also found but to a lesser extent. **(C)** Venn diagram of azole resistance mechanisms in serial clinical isolates and in *in–vitro* evolution in *C neoformans* and *C gattii* isolates. Aneuploidy, upregulation of efflux pumps, upregulation and mutations in ERG11, and CNVs are common to both evolutionary tracks.

As stated above, in *C. neoformans*, the main resistance mechanism activated in response to prolonged triazole exposure is through the generation of hetero–resistance and aneuploidies. Indeed, several studies analyzing serial clinical isolates, identified key aneuploidies in Chr1 (encoding *AFR1* (an ABC transporter) and *ERG11*), Chr12 (encoding several oxidative stress related genes, such as dehydrogenases), occurring in that order, and Chr4, Chr5, as well as one deletion in Chr3 (Mondon et al., 1999; Ormerod et al., 2013; Chen et al., 2017; Stone et al., 2019), for which the timeline of occurrence is unknown (**Table 4**; **Figure 3A**). In addition, a correlation between hetero–resistance and Chr1 aneuploidy was found, with reversion of said aneuploidy in the absence of the triazole, suggesting that aneuploids bear a fitness cost (Stone et al., 2019). Even though *ERG11* mutations are not a significant resistance mechanism in *C. neoformans*, an Erg11 G484S mutation was found in a series of clinical isolates (Rodero et al., 2003), corresponding with the known G464S gain–of–function mutation resistance mutation in CaErg11. A third, less documented, resistance mechanism includes the deletion mutation in an ARID (AT–rich interaction domain)–containing gene = *AVC1*, a regulator of virulence traits and carbon assimilation (Ormerod et al., 2013, Chen et al., 2017). However, the resistance mechanism has not been found.

**Table 4:** Development of triazole resistance in serial clinical isolates of *C. neoformans*.

Species	Isolate source	Approach used	Main findings	Reference
*C. neoformans*	Serial clinical isolates from AIDS patients with cryptococcal meningitis	DNA fingerprinting, random amplified polymorphic DNA (RAPD) analysis.	Patient A was infected with two unrelated strains in two episodes of cryptococcal meningitis, in patient B a single persistent strain was responsible for both episodes. Minor polymorphisms were found in the isolates collected within the same episodes.	(Sullivan et al., 1996)
*C. neoformans*	Serial clinical isolates from an AIDS patient	CHEF karyotype analysis, RAPD analysis.	Hetero–resistance to fluconazole and voriconazole. Resistance is influenced by incubation temperature, but not by pH or osmolarity of the medium.	(Mondon et al., 1999)
*C. neoformans*	Serial clinical isolates from an AIDS patient	Sequence of *ERG11.*	No cross–resistance with other triazoles, G484S resistance mutation in Erg11 (corresponding with the known G464S resistance mutation in CaErg11​).	(Rodero et al., 2003)
*C. neoformans*	Serial clinical isolates from an HIV positive patient	WGS, nematode and murine survival assays,	Aneuploidy of Chr12 (encoding several oxidative stress related genes, such as dehydrogenases). Single mutation in an ARID (AT–rich interaction domain)–containing gene = *AVC1*, regulator of virulence traits and carbon assimilation. Suggested involvement of *AVC1* in disease persistence or relapse.	(Ormerod et al., 2013)
*C. neoformans*	Serial clinical isolates from an HIV positive patient	Growth curve, survival assay in *Galleria mellonella.*	Slower growth and decreased virulence of the resistant isolate in 37°C, lower degree of phagocytosis *in–vivo*.	(Rossi et al., 2016)
*C. neoformans*	Serial clinical isolates from AIDS patients (Van Wyk et al., 2014)	WGS, *Galleria mellonella* infections.	Mutations or indels found in: *CRZ1, eIF2A, MCM2, CTK1, RPD3* and *MLH3*. Deletion in Chr3 (containing oxygenase, Cas33 lipase, methyltransferase and ARID domain protein), duplication in Chr5 (containing *SET101, UFD4*, deacetylases, *PMC1*, *HAPX* and *FRE3*). Duplication of *ERG11* and *CRG2*.	(Chen et al., 2017)
*C. neoformans*	Serial clinical isolates from HIV positive patients	R6G efflux assay, RT–PCR of *AFR1* (an ABC transporter) and *ERG11*, WGS.	Hetero–resistance detected in the first–cultured strains, aneuploidies in Chr1, Chr4, and Chr12. Correlation between hetero–resistance with Chr1 disomy and efflux activity, overexpression of *AFR1* and *ERG11* in a hetero–resistant isolate with Chr1 disomy. Reversion of aneuploidy in the absence of drug stress.	(Stone et al., 2019)

#### Experimental evolution in *Cryptococcus* spp. supports mechanisms observed in serial clinical isolate studies

Similarly to the results described in serial clinical isolate studies, aneuploidies are also described in the few triazole resistance evolutionary studies performed in *C. neoformans* (Sionov et al., 2010), with the same loss of aneuploidy in the absence of triazole stress (**Table 5**; **Figure 3B**). More specifically, disomy of Chr1 (encoding *AFR1* and *ERG11*), Chr4, Chr10 and Chr14 were identified (Sionov et al., 2010). Overexpression of key genes (*ERG11*, *AFR1* and *MDR1* in *C. neoformans*, and *ERG11* in *C. gattii*) were also described (Bastos et al., 2017), although they were not linked to aneuploidies. As found in the serial clinical isolate studies, one experimental evolution study linked a novel G344S mutation in *ERG*11 to voriconazole resistance (Kano et al., 2017).

**Table 5:** Development of triazole resistance in *Cryptococcus* spp. isolates undergoing *in–vitro* evolution.

Species	Isolate source	Evolution method	Total number of passages	Approach used	Main findings	Reference
*C. neoformans*	Environmental and reference strains	Stepwise increases of **fluconazole**.	NA	Microarray, q–PCR of genomic DNA, gene manipulation.	Fluconazole acquired resistance is partially reversible. Upregulation of genes located mainly on Chr1 and Chr4 due to duplication of these chromosomes. Loss of Chr1 disomy in the absence of triazole stress. The extent of triazole resistance correlates with the number of disomic chromosomes (Chr1, Chr4, Chr10 and Chr14).	(Sionov et al., 2010)
*C. neoformans*	Feline clinical isolate	Passages in increasing or constant concentrations of **voriconazole** on agar plates.	3	Sequencing of *ERG11*, qRT–PCR of *ERG11*, *AFR1*, and multidrug efflux pump–encoding (MEP) genes.	Triazole cross–resistance (fluconazole, itraconazole, voriconazole), G344S mutation in Erg11, no overexpression of the genes tested, synergistic effect of combination of voriconazole and efflux blocker FK506.	(Kano et al., 2017)
*C. neoformans*	Clinical and environmental strains	Stepwise increases of the agricultural triazole **tebuconazole** in broth.	NA	Virulence, and cross–resistance testing in mice, RT–PCR of *AFR1* and *MDR1*.	Triazole cross–resistance (tebuconazole, fluconazole, ravuconazole and in some backgrounds itraconazole) *in–vitro* and *in–vivo*, morphological changes, decreased virulence. *ERG11, AFR1* and *MDR1* overexpression.	(Bastos et al., 2017)
*C. gattii*	Clinical and reference strains	Stepwise increases of the agricultural triazole **tebuconazole** in broth.	NA	Virulence, and cross–resistance testing in mice, RT–PCR of *PDR1* and *MDR1*.	Triazole cross–resistance (tebuconazole, fluconazole, ravuconazole and in some backgrounds itraconazole) *in–vitro* and *in–vivo*, morphological changes, decreased virulence. *ERG11* overexpression.	(Bastos et al., 2017)

### A. fumigatus

Aspergillus species known to cause diseases in humans are mainly *A. fumigatus*, *A. flavus*, *A. niger*, *and A. terreus* (Ben-Ami and Kontoyiannis, 2021). This section will focus on *A. fumigatus*, the primary pathogen in this group (Latgé and Chamilos, 2019). *A. fumigatus* is innately resistant to fluconazole (Leonardelli et al., 2016), therefore the triazoles in use in the clinic today are voriconazole, itraconazole, posaconazole, and isavuconazole. We will describe studies involving these clinical triazoles, as well as the agricultural triazole tebuconazole.

#### Serial clinical isolate studies in *A. fumigatus* confirm previous findings demonstrating frequent mutation of *cyp51A* as a driver for resistance

Serial clinical isolates are usually collected from patients with prolonged disease, such as chronic pulmonary aspergillosis, where there is sufficient time to develop increasing resistance and collect the evolving strains. The number of studies where serial clinical isolates originated from invasive aspergillosis is relatively low as the disease is rapid and does not allow sufficient time for evolution (**Table 6**).

**Table 6:** Development of triazole resistance in serial clinical isolates of *A. fumigatus*.

Species	Isolate source	Approach used	Main findings	Reference
*A. fumigatus*	Serial clinical isolates from a patient with aspergilloma	RAPD analysis, sequencing of *cyp51A*, qRT–PCR of *cyp51A*, *atrF*, *MDR1*, *MDR2*, *MDR3* and *MDR4*.	*Cyp51A* gain–of–function mutations found: M220I first, replaced by G54R later. Overexpression of MDR3, MDR2 and MDR4 observed in earlier isolates, but not in the final isolate.	(Chen et al., 2005)
*A. fumigatus*	Serial clinical isolates from several patients	Sequencing of *cyp51A*.	Cyp51A gain–of–function mutations found: M220K, G54E, G54R, G448S.	(Howard et al., 2009)
*A. fumigatus*	Serial clinical isolates from a patient with aspergilloma	Sequencing of *cyp51A*.	Cyp51A G448S gain–of–function mutation, found in the last, most resistant isolate. Cross–resistance to voriconazole and itraconazole.	(Bellete et al., 2010)
*A. fumigatus*	Serial clinical isolates from a patient with CGD	Sequencing of *cyp51A*, RT–PCR of *cyp51A*, invasive aspergillosis mouse model.	Overexpression of *cyp51A*, reduced growth rate with no reduction in virulence.	(Arendrup et al., 2010)
*A. fumigatus*	Serial clinical isolates from patients with aspergilloma or chronic cavitary pulmonary aspergillosis (CCPA)	Sequencing of *cyp51A*.	Cyp51A gain–of–function mutations found: G54E, G54W, G54R.	(Tashiro et al., 2012)
*A. fumigatus*	Serial clinical isolates from a patient with CGD (Isolates taken from Arendrup et al., 2010)	WGS, sequencing of prioritized genes, RT–PCR of *cyp51A*, testing mutation importance by transformation of susceptible *Af*.	Mutations suspected to be involved in triazole resistance found in: *Erg6, Erg25 HapE*, and three more putative proteins. HapE P88L substitution results in overexpression of *cyp51A* and in itraconazole resistance and voriconazole and posaconazole intermediate resistance.	(Camps et al., 2012)
*A. fumigatus*	Serial clinical isolates from a patient with aspergilloma	Sequencing of *cyp51A*, Cyp51A homology model, testing mutation importance by transformation of susceptible *Af*.	Cross–resistance to itraconazole, voriconazole and posaconazole. *Cyp51A* gain–of–function mutations found: P216L, F219I. *Cyp51A* mutations located close to the opening of one of the two ligand access channel.	(Camps et al., 2012)
*A. fumigatus*	Serial clinical isolates from patients with aspergilloma or invasive pulmonary aspergillosis (IPA)	WGS, sequencing of prioritized genes.	Mutations suspected to be involved in triazole resistance found in: *Cyp51A* (P216L), and several more genes.	(Hagiwara et al., 2014)
*A. fumigatus*	Serial clinical isolates from a single patient	Sequencing of *cyp51A*.	*Cyp51A* gain–of–function mutations found: TR_46_/Y121F/T289A.	(Lavergne et al., 2015)
*A. fumigatus*	Serial clinical isolates from a patient with invasive aspergillosis (IA) and aspergilloma	WGS, *Galleria mellonella* virulence assays.	*Cyp51A* gain–of–function mutations found: G54R, G54V, P216L, M220R. Several SNPs in different genes. Reduced virulence in some resistant isolates.	(Ballard et al., 2018)
*A. fumigatus*	Serial clinical isolates from several patients	Sequencing of *cyp51A*, heterokaryon–compatibility testing.	Mutations found in: *Cyp51A* (F291I, G54E) and *HapE* (P88L).	(Zhang et al., 2019)
*A. fumigatus*	Serial clinical isolates from a patient with chronic pulmonary aspergillosis (CPA)	Sequencing of *cyp51A, g*rowth assay, biofilm assay, *Galleria mellonella* virulence assay, WGS, qRT–PCR of *cyp51A*.	Impaired growth, biofilm formation and virulence. Overexpression of *cyp51A*, *HapE* splice site mutation, several SNP in different genes.	(Ito et al., 2021)

**Figure 4:**
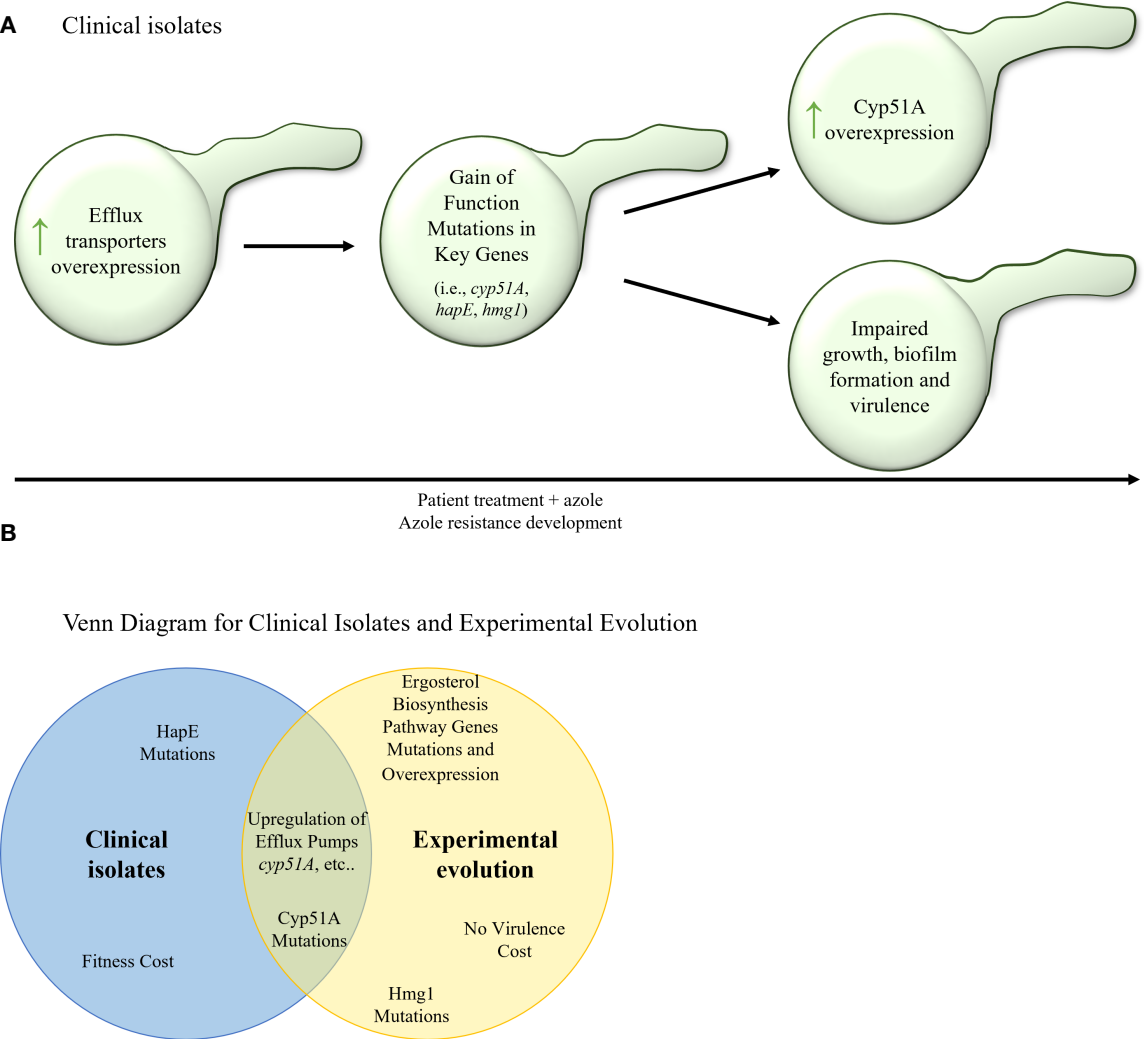
**(A)** Stepwise evolution of triazole resistance acquisition in serial clinical isolates of *A. fumigatus*. An increase of efflux transporter activity is followed by gain–of– function mutations in key genes (*cyp51A*, *hapE*, *hmg1*) and overexpression of key genes (such as *cyp51A* and other *erg* genes). In many cases, these genetic alterations can carry a fitness cost and cause impaired fungal growth, biofilm formation, and virulence. **(B)** Venn diagram of azole resistance mechanisms in serial clinical isolates and in *in–vitro* evolution in isolates of *A fumigatus*. Upregulation of efflux pumps and upregulation and mutations in Cyp51A are common to both evolutionary tracks.

In agreement with what is long known concerning the acquisition of triazole resistance in *A. fumigatus*, serial clinical isolate studies repeatedly document mutation in *cyp51A*, the target enzyme of triazole, which includes the known mutations G54E/R/V/W (Chen et al., 2005; Arendrup et al., 2010; Lavergne et al., 2015; Ballard et al., 2018; Rybak et al., 2019), M220I/K/R (Chen et al., 2005; Lavergne et al., 2015; Rybak et al., 2019), P216L (Camps et al., 2012; Camps et al., 2012; Lavergne et al., 2015), F219I (Camps et al., 2012), G448S (Chen et al., 2005; Howard et al., 2009) and TR_46_/Y121F/T289A (Hagiwara et al., 2014) (**Table 6**; **Figure 4A**). Cyp51A mutations P216L and F219I were proven to cause resistance to itraconazole and posaconazole, and were located close to the opening of one of the two ligand access channels in Cyp51A. Overexpression of *cyp51A* was also observed in several studies (Bellete et al., 2010; Tashiro et al., 2012; Hagiwara et al., 2014), which in some cases can be explained by a mutation in the *cyp51A* transcriptional repressor, HapE (Tashiro et al., 2012; Hagiwara et al., 2014; Ballard et al., 2018), or from a tandem repeat (TR) mutation in the *cyp51A* promoter (Hagiwara et al., 2014), which increases binding of the genes transcriptional activator, SrbA (Nywening et al., 2020). From the studies described here, it appears that overexpression of efflux transporters leading to intermediate resistance is the first step towards triazole resistance acquisition (Rybak et al., 2019). Subsequently *cyp51A* resistance mutations and *HapE* gain–of–function mutations can occur (Chen et al., 2005; Howard et al., 2009; Arendrup et al., 2010; Camps et al., 2012; Camps et al., 2012; Hagiwara et al., 2014; Lavergne et al., 2015; Ballard et al., 2018; Rybak et al., 2019, Tashiro et al., 2012; Zhang et al., 2019; Ito et al., 2021), which in turn can elevate expression of other key genes such as *cyp51A* (**Figure 4A**). These mutations and overexpression can, in many cases, come at a fitness cost, resulting in impaired growth, biofilm formation, and virulence (Bellete et al., 2010; Tashiro et al., 2012; Hagiwara et al., 2014; Lavergne et al., 2015).

#### Experimental evolution studies in *A. fumigatus* agree with findings in serial and non–serial clinical isolate studies

As expected, most *in–vitro* evolution studies discovered cyp51A resistance mutations in the evolved strains, including the known mutations G54E/R/W (da Silva et al., 2004; Losada et al., 2015; Chen et al., 2020), M220I/K/R (Zhang et al., 2019; Chen et al., 2020), G138S (Zhang et al., 2017), and the novel mutations N248K/V436A, Y433N (Chen et al., 2020) (**Table 7**). In addition, *hmg1* mutations that cause resistance in non–serial clinical isolates (Rybak et al., 2019), were also described (Losada et al., 2015; Zhang et al., 2017). Interestingly, but not surprisingly, mutations were also found in other genes of the ergosterol biosynthesis pathway (da Silva Ferreira et al., 2004, Losada et al., 2015), as well as overexpression (da Silva Ferreira et al., 2004; Aruanno et al., 2021), but were not proven to confer triazole resistance directly. Another similarity to what is known from clinical isolates is the correlation between triazole resistance and overexpression of efflux transporters observed in several experimental evolution studies (da Silva Ferreira et al., 2004; Aruanno et al., 2021). It is worth noting that in the studies performed with agricultural triazoles, cross resistance with the medical triazoles itraconazole, posaconazole and voriconazole was also seen (Toyotome et al., 2021), suggesting that the use of triazoles in agriculture can give rise to triazole–resistant strains that infect patients. Curiously, while serial clinical isolates often show impaired virulence, isolates from experimental evolution do not (Chen et al., 2020). Overall, it seems that the evolutionary studies repeat the findings of serial clinical isolate studies, although not fully duplicating the timeline suggested above, as *cyp51A* or *hmg1* mutations, for example, can occur early in the experimental evolutionary process at the same time in which enhanced expression of efflux transporters takes place (da Silva Ferreira et al., 2004; Zhang et al., 2017) (**Figure 4B**).

**Table 7:** Development of triazole resistance in *A. fumigatus* isolates undergoing *in–vitro* evolution.

Species	Isolate source	Evolution method	Total number of passages	Approach used	Main findings	Reference
*A. fumigatus*	CEA17 (pyrG mutant)	Stepwise increases of **itraconazole** on agar plates	10	RT–PCR and sequencing of *cyp51A* and *cyp51B*, sterol quantification, testing mutation importance by transformation of susceptible *Af*, RT–PCR of *MDR1*, MD*R2, MDR4*, *AtrF MDR3*.	Less ergosterol biosynthesis inhibition in the presence of itraconazole. Cyp51A gain–of –function mutations found: N22D, G54R and M220I. Cyp51B mutations found: F59L, S177F, P178P and S505P. Different patterns of increased and decreased expression of *cyp51A* and efflux transporters in different isolates.	(da Silva Ferreira et al., 2004)
*A. fumigatus*	Clinical, environmental and laboratory strains	Stepwise increases of **itraconazole, posaconazole,** or **voriconazole** on agar plates	NA	WGS, crossing of isogenic mating types to test mutation importance.	Mutations in Cyp51A (G54R), GanA (transcriptional activator) and an ABC transporter were found in resistant isolates evolved with all three triazoles, Hmg1 (E307D) mutations were found only in voriconazole–evolved strains, Erg25A and Ssc70 (stress response chaperone) mutations were found only in itraconazole–evolved strains.	(Losada et al., 2015)
*A. fumigatus*	Environmental strain	Passages in constant concentrations of **bromuconazole, tebuconazole, epoxiconazole, difenoconazole,** or **propiconazole** on agar plates.	7	Mycelial growth rate measurement, MIC. WGS, evolutionary mutation tracking.	Asexual sporulation is required for the development of triazole resistance. Cross resistance with the medical triazoles itraconazole, posaconazole and voriconazole. Mutations found: Hmg1 P320L, Cyp51A G138S, PtaB Q264STOP.	(Zhang et al., 2015), (Zhang et al., 2017)
*A. fumigatus*	Clinical strains	Stepwise increases of **itraconazole,** on agar plates	6/17	*Cyp51A* sequencing and 3D structural model analysis, testing mutation importance by transformation into susceptible *Af*. *Galleria mellonella* virulence assay.	Cyp51A gain–of–function mutations found: N248K/V436A, Y433N, M220I/K/R, G54E/W. No reduced virulence.	(Chen et al., 2020)
*A. fumigatus*	Laboratory strain	Stepwise increases of **tebuconazole,** on agar plates	3	WGS, testing mutation importance by transformation into susceptible *Af*, qPCR of *cyp51A*.	Mutations found in: MfsD (R337L), AgcA (E535Stop), UbcD (T98K), AbcJ (G297E), RttA (A83T). Cross resistance with the medical triazoles itraconazole, posaconazole and voriconazole.	(Toyotome et al., 2021)
*A. fumigatus*	Laboratory strain	Stepwise increases of **voriconazole,** on agar plates.	10	Sequencing of *cyp51A*, *hmg1, hapE, atrR*, and *srbA*. RNAseq, RT–PCR, analysis of sterol components by mass spectrometry, measurement of transporter activity by rhodamine 6 G.	Overexpression of *cyp51A, erg1, erg3, erg3A, erg24, erg24B, erg25, erg25B, abcB, abcD, mdr1, mfsC, mdrA* and *asg1*. Decreased ergosterol biosynthesis inhibition in the presence of voriconazole. Deletion of transcription factor Asg1 affects expression of efflux transporters, but does not change triazoles MIC.	(Aruanno et al., 2021)

## Conclusions

**Figure 5:**
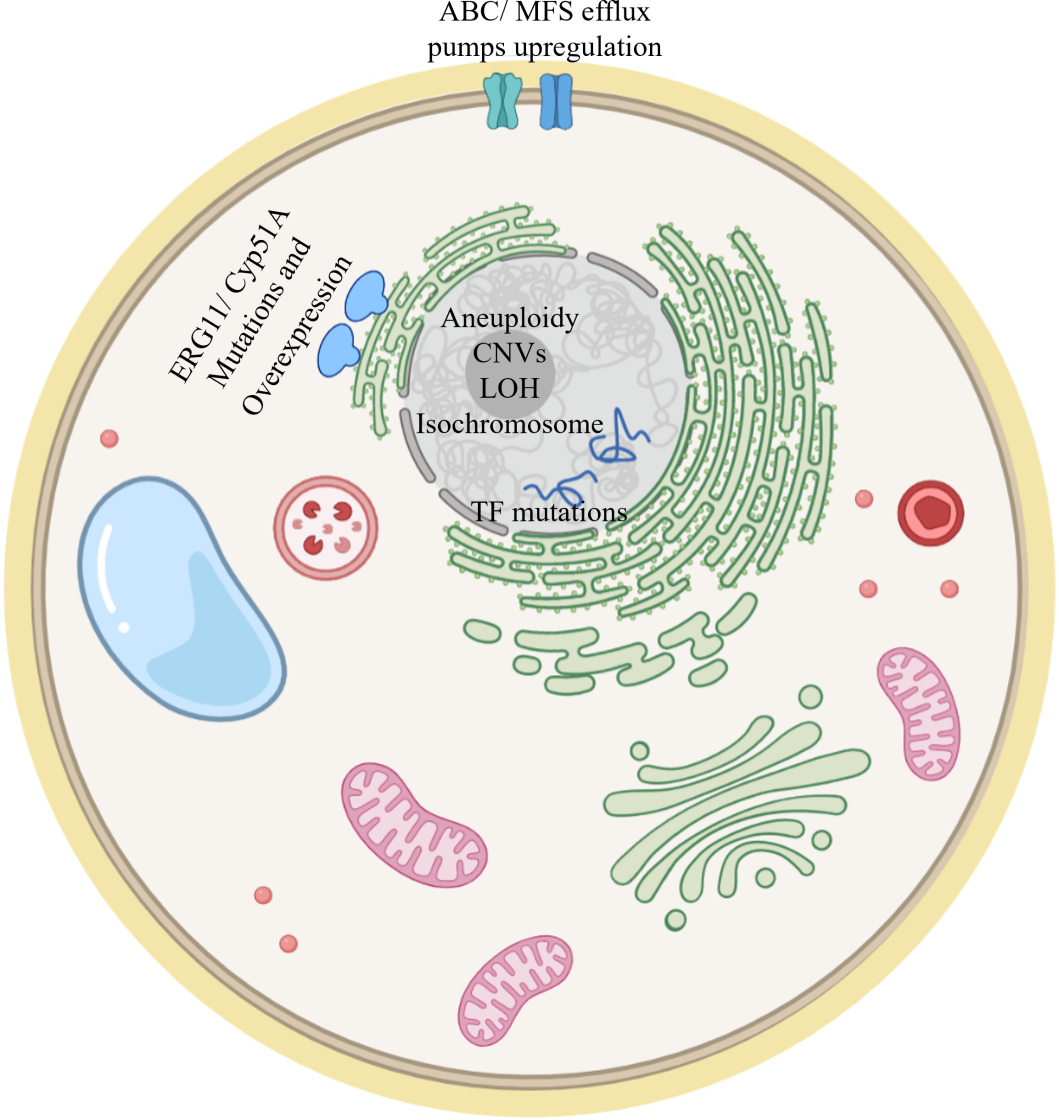
Model of a fungal cell showing the main resistance mechanisms described in this review. ERG11/Cyp51A mutation and overexpression have been documented in *Candida spp*, *Cryptococcus spp* and *A. fumigatus*. Upregulation of efflux pumps has also been described in both clinical and experimental evolution studies for the three fungi addressed in this review. Mutations in transcription factors (TFs) modulating *ERG11*/*cyp51A* gene expression have been described. Aneuploidy, CNVs, LOH and isochromosome formation have been documented in *Candida spp* and *Cryptococcus spp*.

This article reviewed studies describing the development of fungal triazole resistance. They were performed either by “natural” evolution in infected patients resulting in serial clinical isolates, or by experimental evolution under controlled laboratory conditions. The main resistance mechanisms described in this review are visualized in **Figure 5**. In general, resistance mechanisms identified by experimental evolution match the results seen in serial clinical isolates. For example, in all three organisms reviewed here, key shared drivers of resistance are the overexpression of *ERG11/cyp51A*, encoding the target enzyme of triazoles, and of efflux transporters from the ABC (*CDR1/2* in *Candida* spp., *AFR1* in *Cryptococcus* spp., *abcB/D* in *A. fumigatus*) and MFS (*MDR1* in *Candida* spp. and *Cryptococcus* spp., *mdrA* in *A. fumigatus*) families. Likewise, gain–of–function mutations, mainly in the *ERG11/cyp51A* gene, resulting in alteration of the interaction with triazoles, can be found in serial clinical and experimental resistant isolates of both *A. fumigatus* and *Candida* spp., and in experimental resistant isolates of *Cryptococcus* spp. Similarities between *Candida* spp. and *C. neoformans* can be found in aneuploidy and CNVs mechanisms, found in both approaches in both yeasts (Mondon et al., 1999; Coste et al., 2007; Selmecki et al., 2008; Sionov et al., 2010; Huang et al., 2011; Ormerod et al., 2013; Chen et al., 2017; Stone et al., 2019; Todd et al., 2019; Todd and Selmecki, 2020; Carolus et al., 2021; Kukurudz et al., 2022). Aneuploidies have not been reported in *A. fumigatus.* They are unlikely to be an triazole resistance mechanism in this organism, possibly due to its multinuclear filamentous structure, which dilutes the effects of aneuploidy. One major feature differentiating *Cryptococcus neoformans* from *A. fumigatus* and *Candida* spp. is that transient aneuploidy is the main triazole–coping mechanism in *Cryptococcus* (Sionov et al., 2010; Stone et al., 2019).

In serial clinical isolates of *Candida* spp., mutations in transcription factors Upc2, Tac1 and Mrr1 occur and then lead to increased expression of key genes such as *ERG11* and efflux transporters, while in *A. fumigatus* increased efflux transporter expression precedes gene mutations in serial clinical isolates. A sequence of events was not suggested for experimental evolution isolates of *Candida* spp. In *Cryptococcus* spp., serial clinical and experimental evolutionary isolates aneuploidies and CNVs lead to elevated expression of the key genes mentioned above.

The study of serial clinical isolates and experimental evolution studies yielded valuable information, including novel mutations found in *C. neoformans* (Erg11 G344S, Kano et al., 2017) and *A. fumigatus* (HapE P88L, Camps et al., 2012), and CNVs in *C. albicans* (Todd and Selmecki, 2020).

In summary, experimental evolution studies of triazole resistance in pathogenic fungi are valuable tools for determining the mechanisms of resistance acquisition. Despite not replicating the patient environment, they repeat the main findings from serial clinical isolates. They also go beyond them in their ability to monitor the timeline of changes occurring during triazole exposure in proven isogenic strains. Recently, with the advent of omics tools and CRISPR–Cas9 editing technology, the full value of experimental evolution studies has become more evident. This includes the ability to sequence intermediate isolates to precisely deduce the timeline of resistance acquisition, the use of CRISPR–Cas9 editing technology to more rapidly analyze the contribution of mutations in non–coding regions, hypothetical genes and in multiple gene combinations, and the evolution of mutant strains that can highlight alternative pathways of evolution.

## Author contributions

All authors listed have made a substantial, direct, and intellectual contribution to the work and approved it for publication.

## Funding

This review was supported by the Israel Science Foundation (ISF) grant 2444/18 to NO.

## Conflict of interest

The authors declare that the research was conducted in the absence of any commercial or financial relationships that could be construed as a potential conflict of interest.

## Publisher’s note

All claims expressed in this article are solely those of the authors and do not necessarily represent those of their affiliated organizations, or those of the publisher, the editors and the reviewers. Any product that may be evaluated in this article, or claim that may be made by its manufacturer, is not guaranteed or endorsed by the publisher.
